# A Global–Local Attention Model for 3D Point Cloud Segmentation in Intraoral Scanning: A Novel Approach

**DOI:** 10.3390/bioengineering12050507

**Published:** 2025-05-11

**Authors:** Haiwen Chen, Yuan Qin, Baoning Liu, Houzhuo Luo, Ruyue Qiang, Yanni Meng, Zhi Liu, Yanning Ma, Zuolin Jin

**Affiliations:** 1 State Key Laboratory of Oral & Maxillofacial Reconstruction and Regeneration, National Clinical Research Center for Oral Diseases, Shaanxi Clinical Research Center for Oral Diseases, Department of Orthodontics, School of Stomatology, The Fourth Military Medical University, Xi’an 710032, China; 2 School of Information Science and Engineering, Shandong University, Qingdao 266237, China; 3 Department of Anesthesiology, Shaanxi Provincial People’s Hospital, Xi’an 710068, China

**Keywords:** orthodontics, optical imaging, deep learning, image processing, computer-assisted

## Abstract

**Objective:** Intraoral scanners (IOS) provide high-precision 3D data of teeth and gingiva, critical for personalized orthodontic diagnosis and treatment planning. However, traditional segmentation methods exhibit reduced performance with complex dental structures, such as crowded, missing, or irregular teeth, constraining their clinical applicability. This study aims to develop an advanced 3D point cloud segmentation model to enhance the automated processing of IOS data in intricate orthodontic scenarios. **Methods:** A 3D point cloud segmentation model was developed, incorporating relative coordinate encoding, Transformer-based self-attention, and attention pooling mechanisms. This design optimizes the extraction of local geometric features and long-range dependencies while maintaining a balance between segmentation accuracy and computational efficiency. Training and evaluation were conducted using internal and external orthodontic datasets. **Results:** The model achieved a mean Intersection over Union (IoU) of 92.14% on the internal dataset and 91.73% on the external dataset, with Dice coefficients consistently surpassing those of established models, including PointNet++, TSGCN, and PointTransformer, demonstrating superior segmentation accuracy and robust generalization. **Conclusions:** The model significantly enhances tooth segmentation accuracy in complex orthodontic scenarios, such as crowded or irregular dentitions, enabling orthodontists to formulate treatment plans and simulate outcomes with greater precision—for example, optimizing clear aligner design or improving tooth arrangement efficiency. Its computational efficiency supports clinical applicability without excessive resource consumption. However, due to the limited sample size and potential influences from advancements in IOS technology, the model’s generalizability requires further clinical testing and optimization in real-world orthodontic settings.

## 1. Introduction

Intraoral scanning (IOS) technology, which relies on high-precision optical sensors to capture three-dimensional (3D) surface data of teeth and gingiva, has emerged as an essential tool in orthodontics, restorative dentistry, and implantology [1,2]. In relation to orthodontic treatment, IOS technology serves a crucial role throughout the entire treatment process, from initial diagnosis to posttreatment assessment, by providing clinicians with precise 3D data. However, the raw IOS data consist of detailed 3D surface models of the oral cavity, and their direct application poses challenges for further treatment analysis and planning. The accurate segmentation of teeth and gingiva remains essential for personalized treatment planning, clear aligner design, and treatment progress evaluation [3].

While IOS sensors capture highly precise surface geometries, they record only external surface information. This complicates segmentation tasks requiring higher precision due to complex tooth arrangements, crowding, and missing teeth. Noise, occlusion, and incomplete surface information during acquisition may also adversely affect segmentation performance. Traditional manual segmentation approaches and rule-based automated methods are inadequate for addressing these conditions [4].

This indicates that the efficient automation of segmentation algorithms is essential for enhancing the accuracy of teeth and gingiva segmentation and optimizing data processing workflows in orthodontic treatment.

While traditional approaches exhibit clear limitations, deep learning techniques [5,6,7], recognized for their robust capability in automatically learning feature representations directly from data, have demonstrated superior performance in this field. These advanced computational models provide enhanced accuracy and adaptability in segmenting complex dental structures by directly extracting relevant multi-scale geometric features from the 3D data. Among these methods, approaches processing data as point clouds—collections of 3D coordinate points representing the surface—have gained prominence. PointNet [8] marked the initial advancement, directly processing raw point cloud data and extracting local and global geometric features without the information loss associated with conventional projection or voxelization (converting data into grid-like structures). Subsequently, techniques incorporating hierarchical structures and multi-scale feature learning, such as PointNet++ [9], have improved capabilities for handling complex tooth morphologies. DCNet [10], using concepts from dynamic graph convolutional networks (DGCNNs) [11] which analyze relationships between points, dynamically adjusts the neighborhood structure within the point cloud, improving segmentation precision, particularly at challenging tooth and gingival boundaries. Furthermore, Transformer-based models like TSegFormer [12] employ self-attention mechanisms—allowing the model to weigh the importance of different points—to capture long-range dependencies within the data, significantly improving segmentation performance. These deep learning strategies provide clinicians with potentially more accurate digital representations, forming a better foundation for subsequent clinical tasks like virtual treatment planning and appliance fabrication.

In contrast, mesh-based networks process the triangular mesh data commonly output by scanners (e.g., STL format). MeshSegNet [13] uses multi-scale graphical modules to optimize local feature extraction, demonstrating robust performance, even in complex orthodontic cases. TSGCNet [14], a dual-stream graph network analyzing both point positions and surface orientations (normal vectors), enhances accuracy, especially for intricate tooth boundaries. While view-based [15] (projecting 3D to 2D images) and voxel-based [16] (using 3D grids) methods exist, their clinical application remains limited. View-based methods inherently lose 3D spatial information during projection, potentially compromising the segmentation accuracy needed for precise orthodontic measurements. Voxel-based methods, while preserving spatial characteristics better, often face substantial computational demands, particularly with the high-resolution scans used clinically, limiting their practicality. This landscape highlights the following critical clinical need: balancing the demand for high segmentation accuracy, which directly impacts treatment quality, with the computational efficiency required for practical integration into daily orthodontic workflows.

Despite the significant improvements in performance brought by deep learning methods, several challenges remain, particularly impacting the reliability of segmentation in clinically encountered complex orthodontic cases. Extracting local features remains a substantial challenge. Classic point cloud models, such as PointNet and PointNet++ [17], rely on absolute coordinate representations, which complicate the precise delineation of tooth boundaries in scenarios involving crowding or atypical morphologies due to ambiguous relative spatial relationships. Another critical challenge lies in modeling global dependencies—that is, capturing the relationships among distant points within the dental arch. Orthodontic treatment inherently involves changes spanning the entire dental arch [18]; however, most existing models predominantly focus on local geometric features, inadequately capturing these essential long-range structural relationships. Although graph-based networks attempt to incorporate broader contextual information by leveraging graph structures, they often face difficulties in effectively integrating fine local details with global structural context in cases of severe malalignment or missing teeth [19]. Therefore, the development of frameworks capable of efficiently and flexibly integrating detailed local features and comprehensive global context is essential to accurately model complex tooth relationships in orthodontic scenarios [20], potentially yielding significant improvements in treatment simulation and outcome prediction.

To address these challenges, this study introduces a novel multi-scale feature fusion strategy. It leverages relative coordinate encoding [21]—focusing on the position of points relative to their neighbors—to enhance local feature extraction, particularly in complex arrangements. Concurrently, it incorporates Transformer-based attention mechanisms [22] to effectively establish long-range dependencies across the entire dental arch. Additionally, the proposed model employs an attention pooling mechanism [23] to dynamically weight and integrate these local and global features, ensuring a balanced and context-aware segmentation process. By tackling these specific limitations, this study aims to develop a more accurate and robust segmentation model tailored for orthodontic scenarios, thereby providing clinicians with a more reliable tool for precise personalized treatment planning, simulation, and monitoring.

## 2. Materials and Methods

In this study, a 3D point cloud segmentation model for intraoral scanning (IOS) data is presented, capable of accurately segmenting teeth and gum in complex orthodontic scenarios. Given that the original data format is a mesh, it is first converted to a point cloud representation, and mechanisms are developed to capture the geometric structure of the teeth and the boundary relationships between the teeth and gum. This paper proposes a general architecture with the following three modules: a preprocessing module, a feature extraction module, and a feature fusion module. These modules are responsible for data sampling, feature extraction, and feature fusion and are used to obtain local and global geometric information from point cloud data.

**Figure 1:**
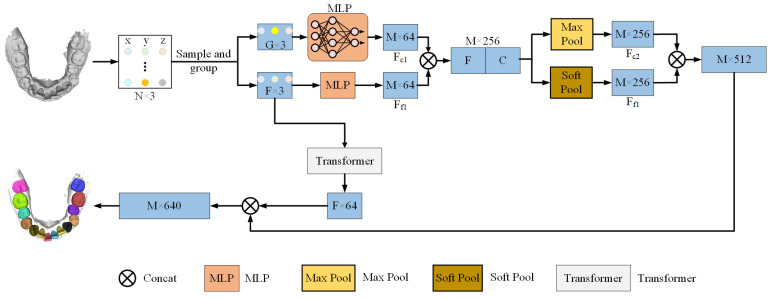
Overall architecture of the proposed point cloud segmentation model.

Figure 1 provides an overview of the model’s operation. In this study, the preprocessing step employs the Farthest Point Sampling (FPS) [24] method to sample and group the point cloud into a reduced set of points, thereby reducing computational complexity while preserving essential geometric information. Then, utilizing relative coordinate encoding, the model calculates the relative positions and distances between each central point and its neighboring points. This enhances the model’s capability to capture local geometric features by providing a more accurate description of spatial relationships for subsequent feature extraction. The feature extraction module employs Multilayer Perceptrons (MLPs) [25] in conjunction with Transformer self-attention mechanisms to capture both local and global geometric features. The feature fusion module then applies an attention pooling mechanism to weight and fuse both local and global geometric features, thereby producing the final segmentation result. In this manner, the model achieves high accuracy in segmenting complex dental geometries and gum boundaries, particularly in complex orthodontic scenarios.

### 2.1. Preprocessing Module

**Figure 2:**
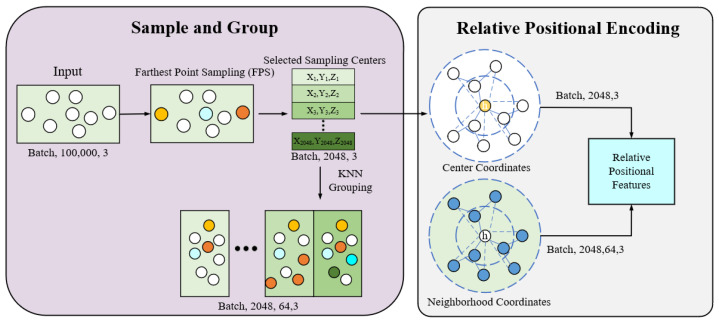
Workflow of the preprocessing module of the proposed model, including sampling, grouping, and relative positional encoding.

The input IOS point cloud data are sampled and grouped in the preprocessing module to reduce the computational burden while preserving important local and global geometric information, as illustrated in Figure 2. Each point cloud has roughly 200,000 points. To balance computational efficiency with the preservation of geometric details, 2048 center points are uniformly sampled from each sample using Farthest Point Sampling (FPS). This number represents a critical trade-off between computational efficiency and segmentation accuracy. While fewer points reduce computational load, sampling too few (e.g., less than 1024) can risk losing fine geometric features critical for accurate segmentation, especially in complex orthodontic scenarios characterized by intricate surface variations. Based on preliminary experiments, sampling 2048 points was found to provide an effective balance. FPS ensures that the sampled points represent the major regions of tooth surfaces, thereby effectively removing redundant data while preserving global geometric features. The k-NN algorithm [26] is then applied to select 64 points around each central point. Specifically, the k-NN algorithm captures local geometric structures, particularly in complex regions such as tooth and gingival boundaries, which is crucial for handling orthodontic scenarios involving irregular dentition or missing teeth. The most important innovation introduced in this model is relative position encoding. Compared to traditional methods that rely solely on absolute coordinates, the use of relative coordinates enhances the model’s ability to capture local geometric structures and provides more precise descriptions of the spatial relationships among points. By computing the relative coordinates within the point cloud, it captures local geometric variations in unordered point clouds, significantly improving segmentation accuracy and robustness. The relative position between the center point and its neighborhood points is defined as follows:

(1) $$r_{ij}=p_{j}-p_{i}$$

where $p_{i}$ and $p_{j}$ represent the three-dimensional coordinates of the center point and the neighborhood points, respectively. To further enhance the model’s understanding of geometric information, the Euclidean distance ($d_{\mathrm{ij}}$) between each neighborhood point and the center point is also calculated, given by the following:

(2) $$d_{ij}=\sqrt{{\left(x_{j}-x_{i}\right)}^{2}+{\left(y_{j}-y_{i}\right)}^{2}+{\left(z_{j}-z_{i}\right)}^{2}}$$

By combining relative position ($r_{\mathrm{ij}}$) and distance ($d_{\mathrm{ij}}$), the local geometric relationships in the point cloud can be more effectively described, providing essential inputs for subsequent feature extraction steps. Through the integration of farthest point sampling, k-NN grouping, and relative position encoding, the preprocessing module ensures the effective extraction of both local and global geometric information while maintaining optimized computational efficiency.

### 2.2. Feature Extraction Module

**Figure 3:**
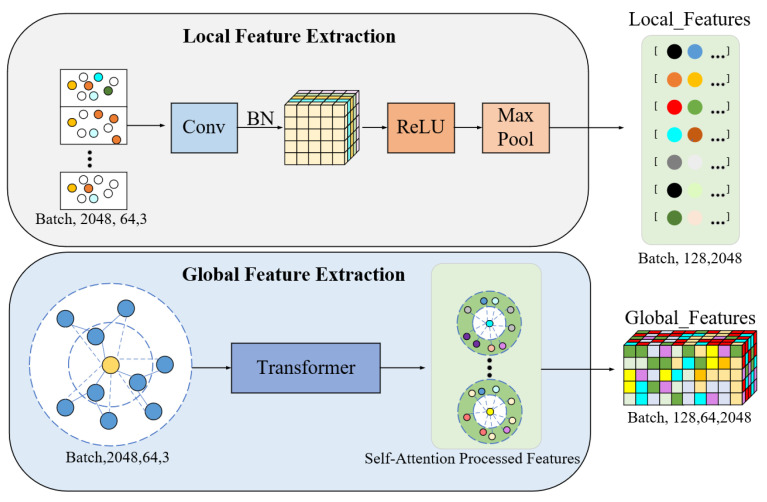
Workflow of the feature extraction module of the proposed model, including local and global feature extraction.

In the feature extraction module (Figure 3), the incorporation of Multilayer Perceptrons (MLPs) together with the self-attention mechanisms of the Transformer in capturing geometric features from the point cloud at both the local and global levels is performed. First, it conducts local feature extraction using MLPs; the relative coordinate information of each sampled point and its neighboring points should be processed. In particular, each point’s relative coordinates are processed through point-wise convolutional networks to generate 128-dimensional local feature vectors layer by layer. The formula is as follows:

(3) $$F_{MLP}=MLP\left(r_{ij}\right)$$

where${\;F}_{\mathrm{MLP}}$ denotes the output local feature vector, and $r_{ij}$ represents the relative coordinates between the center point and its neighboring points. ReLU activation functions and Batch Normalization are introduced in the MLP layers to enhance the model’s training stability and generalization ability. These mechanisms ensure model stability during training and reduce gradient vanishing or explosion issues. Moreover, after processing by the MLP, max pooling is applied along the neighborhood dimension to extract the locally salient features of each center point.

Furthermore, this model provides the Transformer self-attention mechanism to better model global spatial dependencies. The transformer dynamically captures long-range dependencies among pairs of points by the generation of Query, Key, and Value matrices. Concretely, this is carried out by the following:

(4) $$Q=XW_{Q},K=XW_{K},V=XW_{V}$$

Then, by computing the dot product of queries and keys, attention weights A are obtained, which can be expressed as follows:

(5) $$A=softmax\left(\frac{QK^{T}}{\sqrt{d_{\mathrm{model}}}}\right)$$

where $d_{\mathrm{model}}$ is the scaling factor used to prevent numerical values from becoming too large and causing gradient vanishing problems. Subsequently, the attention weights are used to weight the value matrix, obtaining the following weighted feature representation:

(6) Z=AV

In this model, eight multi-head attention mechanisms are employed, with each attention head having a feature dimension of 32, resulting in a total dimension of 256. Multi-head attention integrates features from different perspectives, aiding the model in better capturing global geometric relationships. The local features extracted by the MLP are concatenated with the global features generated by the Transformer through feature fusion. The concatenated feature vector serves as the input to the next feature fusion module, providing more comprehensive geometric information for the model in tooth and gingiva segmentation tasks.

### 2.3. Feature Fusion Module

**Figure 4:**
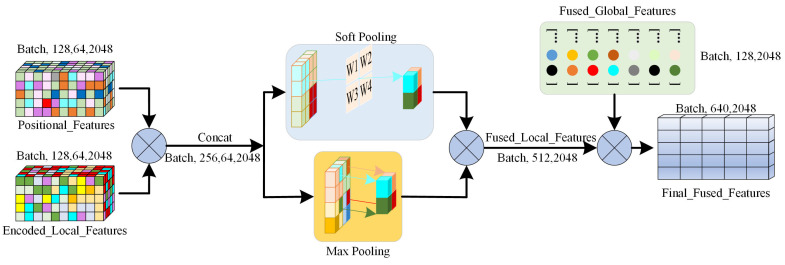
Workflow of the feature fusion module of the proposed model, incorporating attention pooling, max pooling, and soft pooling.

In the feature fusion module (Figure 4), it uses an attention pooling mechanism to combine the local and global features of the model for modeling teeth and gingiva geometric information at different scales. First, there is the linear transformation of each input feature to obtain the attention weight as follows:

(7) $$\alpha_{i}=\frac{\exp\left(Wx_{i}\right)}{\sum_{j}\exp\left(Wx_{j}\right)}$$

where $x_{i}$ represents the i-th feature in the input feature vector, W is the weight matrix of the linear transformation, and $\alpha_{i}$ denotes the weight of the input feature. The weights are normalized using Softmax so that the sum of all weights equals 1, reflecting the relative importance of each feature in the fusion process. Features with higher weights will influence the final feature representation more. Next, these weights are used to derive a weighted sum of the input features to generate the weighted feature representation, given as follows:

(8) $$f_{\mathrm{out}}=\underset{i}{\sum }\alpha_{i}x_{i}$$

where $f_{\mathrm{out}}$ is the output fused feature, obtained by calculating the weighted sum over each feature to generate a composite feature representation.

Subsequently, the model further extracts features from both local and global levels using max pooling and soft pooling, respectively. This process aims to extract the most salient local geometric features that capture essential information about the geometric characteristics of neighboring points. Max pooling is effective at identifying significant geometric changes along the boundary between the teeth and gingiva. In contrast, soft pooling is utilized to aggregate global geometric features by assigning a weight to each feature that reflects its relative importance [27]. This ensures the retention of global information without losing important details within the overall geometric structure. Finally, the model concatenates the local and global features to obtain a comprehensive feature vector, which is expressed as follows:

(9) $$F_{\mathrm{fusion}}=Concat\left(F_{local},F_{global}\right)$$

where $F_{\mathrm{fusion}}$ represents the fused feature vector combining both local and global information, $F_{local}\;$ represents the local features extracted using max pooling, $F_{global}$ represents the global features extracted through soft pooling, and Concat denotes the feature concatenation operation. Through this multi-scale feature fusion mechanism, the model not only captures the local features of teeth and gingiva but also models the global dependencies of the entire dentition.

## 3. Results

### 3.1. Dataset

The dataset includes an intraoral scanner dataset of 894 oral scans collected from two tertiary hospitals and three dental clinics in Shaanxi and Shanxi provinces in China between 2018 and 2023. The patients were aged between 12 and 41 years with permanent dentition, who had undergone bracketless clear aligner treatment. Regarding the source, the dataset is split into an internal dataset and an external test set. The internal dataset consists of data from the two hospitals, while the external test set comprises data from the three dental clinics. The internal dataset comprised scans acquired using intraoral scanners from 3Shape (TRIOS 2, TRIOS 3; Copenhagen, Denmark) and Align Technology (iTero Element 1, iTero Element 5D Plus; San Jose, CA, USA). The external dataset utilized scans from 3Shape (TRIOS 3, TRIOS 4) and Align Technology (iTero Element 2, iTero Element 5D). The internal dataset was divided into training, validation, and test sets in a ratio of 70%, 15%, and 15%, containing 396, 85, and 85 samples, respectively. The external test set includes 328 samples and is primarily used to evaluate the model’s generalization ability. The patients’ gender, age distribution, and dental anomalies are presented in Table 1, ensuring the dataset’s diversity and representativeness.

All data annotations strictly followed the FDI tooth notation system and were labeled using the Semantic Segmentation Editor (SSE) software. Three experienced orthodontists independently completed the annotations and were subsequently reviewed by a senior orthodontic expert to ensure accuracy and consistency. After annotation, all data underwent preprocessing. First, the original point cloud data were aligned to a unified coordinate system. Next, the point cloud coordinates were normalized to adjust the coordinate range to [−1, 1], reducing the impact of data source differences on model training. This study received ethical approval, and all research procedures strictly adhered to the 1975 Declaration of Helsinki and relevant international medical ethics guidelines. All patients’ personal information was anonymized to ensure privacy and security.

### 3.2. Experimental Details

A deep learning-based 3D point cloud segmentation model was employed to handle the panoramic segmentation task of teeth. The model was implemented using the PyTorch 2.1.1 framework. The training process utilized the Adam optimizer with an initial learning rate set to 1 × 10^−4^, a batch size of 16, and a total of 200 training epochs. An early stopping strategy was applied to prevent overfitting. Training was conducted on an NVIDIA RTX 3090Ti GPU (manufacturer: ASUSTeK Computer Inc., sourced from Shanghai, China).

The model uses a cross-entropy loss function to predict class labels accurately for tooth segmentation, measuring the difference between the predicted classification probability of each point and the true label. The cross-entropy loss is more sensitive to classification accuracy than Dice loss, which helps ensure that the model predicts the classification probability of each point with high accuracy. The formula is as follows:

(10) $$L_{\mathrm{CE}}=-\frac{1}{N}\overset{N}{\underset{i=1}{\sum }}\overset{K}{\underset{j=1}{\sum }}y_{i,j}\log\left(p_{i,j}\right)$$

where N is the total number of points, K is the number of classes, $y_{i,j}$ is the true label of the i-th point for class c, and $p_{i,j}$ is the model’s predicted probability for class c. Cross-entropy loss optimizes the model by calculating the difference between each point’s predicted classification probability and its true label.

Data augmentation was extensively used during model training as follows: random rotation, translation, and scaling were used to improve the generalization capability of the model against new data. These augmentations introduce random geometric transformations during training, increasing diversity to help avoid overfitting problems. It particularly transformed the range of the rotation of the point cloud to [−30°, 30°], that of the scaling factor to [0.9, 1.1], and that of translation to [−0.05, 0.05], so that the model could effectively extract features under different transformations.

### 3.3. Model Performance Evaluation and Result Analysis

The Intersection over Union (IoU), Dice coefficient, Overall Accuracy (OA), and Mean Class Accuracy (mAcc) were employed for comprehensive model performance evaluation in panoramic teeth segmentation. These metrics reflect performing segmentation from a different perspective and hence give deeper insight into the effectiveness of the model in dealing with different categories of teeth. IoU is the standard metric in semantic segmentation, which calculates the overlap between predicted regions and ground truth annotations. It exactly measures the boundary precision of segmentation for every category. Similarly to the IoU, the Dice coefficient is indispensable in estimating regional overlap between the predicted and actual regions. It supplements the IoU, reflecting the general quality of overlap in segmentation results and is particularly robust when the regions of categories are smaller. Overall Accuracy reflects the proportion of correctly classified points across the entire dataset, which intuitively makes sense for estimating the global performance of the model on all tooth categories. To better evaluate the model’s consistency across different categories, Mean Class Accuracy calculates the classification accuracy for each category and then takes the average. mAcc effectively balances performance among different categories, making it particularly suitable for scenarios with uneven category distribution and helping to assess the model’s specific performance in each category more clearly.

The calculation formulas for these evaluation metrics are as follows:

(11) $$mIoU=\frac{1}{C}\overset{C}{\underset{c=1}{\sum }}\frac{TP_{c}}{TP_{c}+FN_{c}+FP_{c}}$$

(12) $$OA=\frac{TP}{TP+FP+TN+FN}$$

(13) $$mAcc=\frac{1}{C}\underset{c}{\sum }\frac{TP_{c}}{TP_{c}+FN_{c}}$$

(14) $$mDice=\frac{1}{C}\underset{c}{\sum }\frac{2\times TP_{c}}{FN_{c}+2\times TP_{c}+FP_{c}}$$

where TP, TN, FP, and FN represent true positives, true negatives, false positives, and false negatives in classification, respectively; C represents the total number of categories, which is 33 in this study, including eight types of teeth and gum.

Table 2 presents the model’s segmentation results on the internal dataset and external test set. Overall, the model achieved an OA of 97.82% and a mAcc of 97.07% on the internal dataset, showing strong segmentation performance and balanced accuracy across categories. On the external test set, the model’s OA was 96.89%, and mAcc was 96.10%, indicating that it maintains good generalization ability when handling new data. The mean IoU (mIoU) on the internal and external datasets was 92.14% and 91.73%, respectively, and the mean Dice coefficient (mDice) was 96.39% (internal dataset) and 95.87% (external test set), demonstrating the model’s high overall segmentation accuracy.

Regarding different tooth categories, the model’s segmentation performance varies significantly. The first molar (T6) and second molar (T7) had the best segmentation results, with IoUs of 94.85% and 95.55%, respectively, indicating high segmentation precision on teeth with larger volumes and clear boundaries. In contrast, the central incisor (T1) and lateral incisor (T2) had relatively lower IoUs of 90.02% and 89.87%, respectively, due to the smaller size of anterior teeth and the frequent occurrence of crowding, overlapping, and ectopic positions in the anterior region in orthodontic scenarios, increasing segmentation difficulty. Wisdom teeth (T8) showed the weakest segmentation performance, with an IoU of 86.63%, which may be related to their morphological complexity and positional variability. On the other hand, the performance of the model was a little worse in terms of segmentation when applied to the external test set, showing analogous differences among categories of teeth as obtained in the internal dataset.

**Figure 5:**
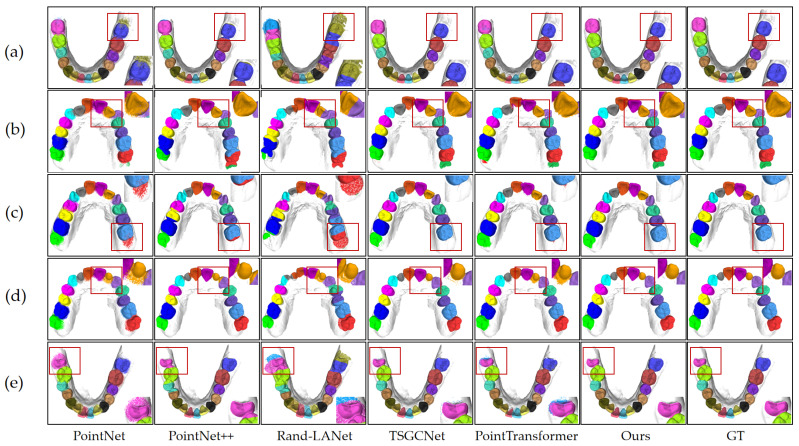
Comparison of tooth segmentation results between five baseline methods, our proposed model, and the ground truth (GT) across different dental scenarios: (**a**) normal dentition, (**b**) crowded teeth, (**c**) missing tooth, (**d**) dental malformations, and (**e**) partially erupted teeth.

The performance of the model was further analyzed across a wide range of clinical orthodontic scenarios based on the segmentation results. Figure 5 illustrates the model’s segmentation effectiveness in five distinct orthodontic situations as follows: normal dentition, crowded teeth, individual tooth loss, partially erupted wisdom teeth, and misaligned teeth. The figure demonstrates that the model accurately identifies tooth positions and precisely segments the boundaries between teeth and between teeth and gums. Thus, the segmentation results remain relatively stable even when faced with challenging dental relationships, demonstrating the model’s applicability in complex orthodontic contexts.

### 3.4. Comparative Experiment Results

In this study, the IOS segmentation task used raw data in mesh format, and researchers have proposed various processing methods, including view-based, voxel-based, point-based, and graph-based strategies. Currently, mainstream methods focus primarily on point cloud [28] and graph processing. To evaluate the performance of the proposed model on this task, five representative baseline segmentation models were selected for comparison as follows: PointNet, PointNet++, RandLA-Net [29], TSGCNet, and PointTransformer [20]. These baseline models represent a mix of classic and emerging techniques in point cloud and mesh data processing, providing a comprehensive evaluation framework for the segmentation of teeth.

PointNet was the first deep learning model to directly process point cloud data, pioneering end-to-end point cloud handling. PointNet++ built on this foundation by incorporating multi-scale feature extraction mechanisms, enhancing its ability to capture local features and improving the precision of point cloud processing. RandLA-Net utilizes random sampling and local aggregation strategies to increase efficiency in handling large-scale point clouds, making it particularly well suited for processing large volumes of data in complex environments. TSGCNet is a dual-stream graph convolutional network that effectively leverages the topological structure of mesh data to process geometric features in triangular mesh format, demonstrating strong performance in prior intraoral dental segmentation tasks. PointTransformer incorporates a self-attention mechanism, excelling at capturing both local and global features, and it has been a significant advancement in point cloud processing in recent years.

Table 3 presents the comparison results between the proposed segmentation model and five baseline segmentation methods for the dental segmentation task. Overall, the proposed model achieved the best performance across all tooth categories. Among the baseline models, TSGCNet performed the best, with an OA of 87.73%, a mIoU of 85.73%, a mDice of 91.26%, and a mAcc of 92.40%. TSGCNet’s strength lies in its effective use of the topological characteristics of mesh data, particularly in addressing complex geometric boundaries and gingival regions. PointTransformer also performed relatively well, achieving an OA of 93.00%, a mIoU of 76.13%, a mDice of 84.19%, and a mAcc of 82.23%. The self-attention mechanism in PointTransformer led to significant improvements in capturing both global and local features, which was especially effective in dealing with complex dental geometries. In contrast, RandLA-Net showed the poorest segmentation results, with an OA of 66.20%, a mIoU of 48.43%, a mDice of 64.90%, and a mAcc of 53.96%. This is likely due to RandLA-Net being primarily designed for efficiency with large-scale point clouds, sacrificing the capability to capture intricate features in complex scenes.

In addition to segmentation results for the overall dataset, a comparison of segmentation results across different types of teeth is provided. Results demonstrate that all baseline models exhibit a similar pattern of distribution to the proposed model, as mentioned earlier. Specifically, higher accuracy in segmentation results for the baseline models was observed for the first and second molars, as these have larger volumes with well-defined boundaries. In contrast, the segmentation of incisors, which are often crowded, sometimes partially overlapped, or partially missing, exhibited lower accuracy. Similarly, all models, including the proposed model, showed reduced accuracy for the complexities introduced by wisdom teeth.

Figure 5 visually compares the segmentation results produced by the proposed model, the ground truth (GT), and the five baseline models. This figure presents the segmentation quality across five different orthodontic scenarios, offering a qualitative complement to the quantitative results. The visual comparison demonstrates the differences among the models in tooth position identification, boundary precision, and capturing details. Compared to the baseline models, the proposed model achieves higher accuracy in tooth position recognition, boundary delineation, and handling complex geometries across various challenging scenarios, consistent with the quantitative metrics. TSGCNet and PointTransformer also demonstrate relatively high segmentation quality; however, certain challenging regions, such as crowded, misaligned, malformed, partially erupted teeth, and areas surrounding missing teeth, still exhibit inaccuracies.

To further evaluate each model’s computational efficiency and resource consumption of each model, Table 4 compares the parameter count, GPU memory usage, and inference time between the baseline models and the proposed model. The results indicate that our proposed model favorably balances performance and computational resources. Although the proposed model has relatively higher GPU memory usage (10.21 G), its inference time is 0.47 s, which is comparable to that of PointNet++ and significantly better than PointTransformer’s 1.01 s.

Regarding parameter count, the proposed model has 3.55 M parameters, significantly fewer than PointTransformer’s 7.8 M, demonstrating its relatively lightweight structure. This allows the model to maintain high segmentation accuracy while reducing computational complexity. By contrast, TSGCN, despite showing good segmentation performance, requires considerably more GPU memory (16.98 G) and a longer inference time (0.59 s).

### 3.5. Ablation Experiment Results

Ablation experiments were designed to evaluate the impact of each improved module on the segmentation task. These experiments were based on PointNet++ as the baseline model and progressively introduced the corresponding improved modules. This section of the study primarily encompasses the following three improvements: introducing relative coordinate encoding to enhance the model’s ability to capture local geometric structures, adopting an attention pooling mechanism to improve the model’s focus on key features through weighted aggregation, and using a Transformer self-attention mechanism to better model global dependencies in the point cloud.

**Figure 6:**
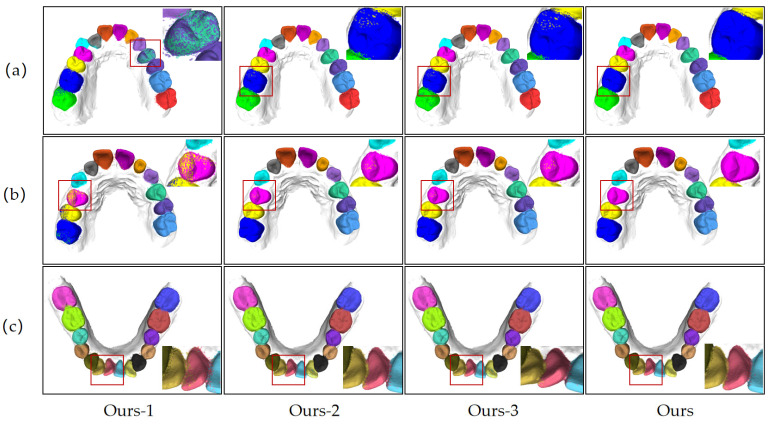
Ablation study on the proposed model under challenging orthodontic scenarios: effects of excluding key components, including relative coordinate encoding (Ours-1), attention pooling (Ours-2), and Transformer self-attention (Ours-3), compared to the complete model (Ours). (**a**) Segmentation results for the upper arch with ectopic teeth, highlighting the effect of different module exclusions on handling positional anomalies; (**b**) segmentation results for scenarios involving microdontia and partially erupted teeth, showing performance variations among different ablated versions when dealing with irregular tooth structures. (**c**) Segmentation results for the crowded lower anterior region illustrate the impact of each excluded component on handling dense and complex tooth arrangements.

For the ablation experiments, this scheme slowly ablates these improvement modules as follows: it removes relative coordinate encoding (Ours-1), removes the attention pooling mechanism (Ours-2), removes the Transformer module (Ours-3), and in the end compares it with the complete model, Ours. The performance for the above-mentioned settings has been measured using OA and mIoU for each experiment to evaluate the overall segmentation performance and also the overlap between predicted and actual regions. Table 5 and Figure 6 show the detailed performance of each improvement module. For instance, without relative coordinate encoding, the model’s OA decreased from 97.82% to 94.67%, and mIoU dropped from 92.19% to 81.71%. This indicates that the module plays a critical role in capturing local geometric features and removing it significantly weakens the model’s capability of grasping the boundaries precisely for some tooth types with either smaller volumes or complex morphologies.

After removing the attention pooling mechanism, Ours-2, the segmentation performance decreased significantly, with an OA of 95.65% and an mIoU of 90.56%, respectively. Compared with the full model, this represents a significant decrease in segmentation performance; hence, it can be concluded that the attention pooling effectively enhances the key feature-capturing ability of the model in weighted feature aggregation. Its elimination leads to deficiencies when the model handles important features, especially in more complex tooth categories. Finally, the Transformer self-attention mechanism was removed to evaluate the performance of Ours-3, which achieved an OA of 94.92% and an mIoU of 87.37%. Since removing the self-attention mechanism weakened the modeling ability of dependencies between distant contexts, the accuracy of the segmentation algorithm decreased compared with the complete model. This demonstrates that the Transformer module significantly improves model performance by modeling global features.

In summary, the results of ablation experiments demonstrate that relative coordinate encoding, the attention pooling mechanism, and the Transformer self-attention mechanism play an important role in improving the local and global feature-capturing ability of the model. From the ablation effects brought on by each module, the results indicate that they are essential for improving segmentation accuracy in complex situations, especially those involving complex tooth boundaries and diverse features.

## 4. Discussion

Contributing mainly to the segmentation of complex IOS data, this paper introduces relative coordinate encoding, an attention pooling mechanism, and Transformer-based self-attention. These innovations significantly enhance the model’s performance in challenging orthodontic scenarios. IOS technology has become an indispensable tool for orthodontics due to its high precision in capturing details on the tooth surface [30]. However, most traditional approaches to tooth segmentation face numerous challenges when addressing difficult morphologies and irregular tooth boundaries. Traditional approaches often rely on equidistant feature extraction or curvature analysis, struggling with malformed teeth, diseased teeth, or significant topological variations. While already achieving considerable success, deep learning methods in general image segmentation are insufficient for handling such complex dentitions and irregular gingival boundaries frequently encountered in orthodontic applications [31,32]. Therefore, improving accuracy for complex geometries while preserving computational efficiency remains a key focus of this study.

This work introduces three major innovations that collectively address these challenges. First, relative coordinate encoding enhances the model’s ability to capture local geometrical features by computing the relative position and distance of each central point to all its neighbors, as shown in [33]. This mechanism provides detailed spatial context, especially for crowded or twisted teeth, thereby improving segmentation accuracy with well-preserved intricate details. Combined with MLPs, it enables accurate feature extraction from complex local structures and provides essential geometric context for subsequent global modeling by the Transformer module.

Based on these local features, the self-attention mechanism within the Transformer enables the model to capture global dependencies from distant points and effectively grasp the overall structure of the dentition [34]. Orthodontic structures often exhibit significant variations in alignment and positioning [35]; thus, their relationships can be better modeled globally. By generating Query, Key, and Value matrices, the Transformer dynamically captures point cloud dependencies, facilitating the more effective integration of global information. Although this mechanism increases the model’s parameter count, the improvement in modeling global features offsets the computational cost by enhancing segmentation performance.

Furthermore, the attention pooling mechanism refines the feature selection process by adaptively selecting key features [36]. The model must identify the most representative features from a large set of complex tooth structures to ensure efficient computation. Attention pooling assigns adaptive weights to features, allowing the model to focus on critical aspects for segmentation while reducing redundancy. This approach improves computational efficiency and ensures precise feature selection, particularly for complex gingival and dental boundaries.

Compared to several established IOS segmentation methods—such as PointNet, PointNet++, RandLA-Net, TSGCNet, and PointTransformer—the proposed model demonstrates significant advantages across multiple aspects. PointNet and PointNet++ are notable for their point-wise processing of point clouds; however, they exhibit limitations in capturing local features, particularly in managing intricate geometries within complex dentitions. TSGCNet and PointTransformer have advanced global feature integration by incorporating geometric and normal vector information, yet they still face challenges with computational efficiency. In contrast, the proposed model leverages relative coordinate encoding combined with a multi-scale feature fusion strategy to effectively integrate global and local features, achieving higher segmentation accuracy, particularly in complex orthodontic scenarios.

The proposed model also achieves a favorable balance between computational efficiency and segmentation performance. Although GPU memory usage is relatively high (10.21 G), the inference time is 0.47 s, which is comparable to that of PointNet++ and notably better than PointTransformer’s 1.01 s. Additionally, compared with PointTransformer’s 7.8 million parameters, the proposed model has a significantly lower parameter count of 3.55 million. This design maintains high segmentation accuracy while reducing excessive computational complexity, making it feasible for practical applications.

While the model offers enhanced segmentation accuracy for complex cases, potentially aiding in treatment planning, its clinical application requires careful consideration. It should function strictly as an assistive tool for qualified dental professionals, not a substitute for their clinical judgment. The interpretation of segmentation results and all subsequent diagnostic and treatment decisions must be made or supervised by trained clinicians. This professional oversight is essential to ensure patient safety, maintain clinical standards, and address the potential ethical and legal considerations inherent in using automated diagnostic aids.

Despite the high performance achieved by this model on some challenging orthodontic cases, several limitations remain. First, the model was trained on a dataset primarily representing common orthodontic complexities. While demonstrating good generalization on external test sets with similar characteristics, its performance on specific, less prevalent dental anomalies (e.g., significant morphological variations and fused teeth) not adequately represented in the training data requires further investigation. Furthermore, the current dataset may lack sufficient diversity across different ethnic groups, which can exhibit subtle variations in tooth morphology and arch forms. Additionally, the dataset spans a six-year collection period (2018–2023) and incorporates data from multiple generations of intraoral scanners. Given the advancements in IOS technology during this time, potential variations in data characteristics across different scanner models and acquisition years represent another limitation. Consequently, future improvements should focus on enhancing generalization by increasing both the scale and explicit diversity (including the broader representation of anomalies, ethnic backgrounds, and potential data stratification by scanner technology) of the dataset. There is also potential for further optimization of inference speed and memory consumption. It is envisioned that the model can be better suited for resource-constrained environments through techniques such as model pruning and quantization, aiming to reduce model complexity without compromising performance.

## 5. Conclusions

This paper proposes an orthodontically complex model for 3D point cloud segmentation with high-precision optical sensing from intraoral scanners. The proposed model can significantly enhance the feature-capturing ability in both local and global aspects by integrating innovative modules, including relative coordinate encoding, Transformer-based self-attention, and an attention pooling mechanism, leading to improved performance in segmenting intricate dentitions and irregular tooth structures. Experimental results have shown that the average IoU against baseline models on both the internal dataset and the external test set reaches 92.14% and 91.73%, respectively. Additionally, other critical metrics describing segmentation precision, such as the Dice coefficient, consistently maintain a high value for the proposed model, thus underscoring its robustness across varying datasets.

Furthermore, the model achieved a really good trade-off between computational efficiency and segmentation performance. Although the memory consumption on the GPU is high, with an inference time of 0.47 s, comparable to the efficient baseline model PointNet++, it significantly improved the accuracy of segmentation with respect to challenging crowded or malformed teeth. This model facilitates the effective and accurate segmentation of teeth and gingiva, thereby providing reliable assistance to dental professionals in formulating more accurate and personalized treatment plans. Further optimization of this model, enabling its use in low-resource settings and extending its application to other optical sensor data, will continue to enhance automation in digital orthodontics and medical imaging.

## Figures and Tables

**Table 1:** Characteristics of the datasets used in this study.

Cohorts	Internal Dataset	External Dataset
Patient number	219	102
Female/male	132/87	75/27
Age	20.5 (13–38)	23.0 (12–41)
IOS number	566	328
Manufacturer	3 Shape/Align	3 Shape/Align

**Table 2:** Segmentation performance of the proposed model on internal and external datasets.

Metric	Internal Dataset		External Dataset	
	IoU	Dice	IoU	Dice
T1	90.02	95.36	89.33	94.63
T2	89.87	96.15	89.11	95.01
T3	92.63	96.15	92.16	95.82
T4	94.37	98.05	93.86	97.20
T5	92.10	97.45	91.76	96.75
T6	94.85	96.99	94.26	96.13
T7	95.55	98.16	94.96	97.36
T8	86.63	93.57	84.23	92.33
Overall				
OA	97.82		96.89	
mAcc	97.07		96.10	
mDice	96.39		95.87	
mIoU	92.14		91.73	

Metrics are shown as percentages (%). T1 (central incisor), T2 (lateral incisor), T3 (canine), T4 (first premolar), T5 (second premolar), T6 (first molar), T7 (second molar), and T8 (third molar).

**Table 3:** Comparison of segmentation performance among different baseline models and the proposed model.

Metric	PointNet	PointNet++	RandLANet	TSGCNet	PointTransformer	Ours
T1	64.27	74.07	57.40	88.16	75.67	90.02
T2	60.94	66.17	50.32	87.81	78.90	89.87
T3	66.02	73.22	56.40	92.02	80.62	92.63
T4	72.98	85.64	57.27	93.30	80.62	94.37
T5	66.16	73.80	58.88	89.46	86.83	92.10
T6	70.90	77.41	47.35	92.80	91.22	94.85
T7	67.93	78.55	44.17	89.90	86.86	95.55
T8	16.75	32.04	10.23	50.23	24.50	86.63
OA	78.40	92.38	66.20	87.73	93.00	97.82
mIoU	61.43	70.80	48.43	85.73	76.13	92.14
mDice	76.97	82.96	64.90	91.26	84.19	96.39
mAcc	65.27	74.27	53.96	92.40	82.23	97.07

Metrics are shown as percentages (%).

**Table 4:** Comparison of model parameters, GPU memory, inference time, and mIoU between baseline and proposed models.

	Para	GPU	Time	mIoU
PointNet	3.53 M	4.39 G	0.42 s	61.34%
PointNet++	0.97 M	5.63 G	0.52 s	70.10%
TSGCNet	4.17 M	16.98 G	0.59 s	85.73%
RandLANet	1.25 M	4.17 G	0.27 s	48.40%
PointTransformer	7.8 M	9.31 G	1.01 s	78.71%
Ours	3.55 M	10.21 G	0.47 s	92.14%

**Table 5:** Ablation study results of the proposed model.

Ablation Module	OA	MIOU
Ours-1	94.67	81.71
Ours-2	95.65	90.56
Ours-3	94.92	87.37
Ours	97.82	92.19

Metrics are shown as percentages (%). Ours-1: without relative coordinate encoding; Ours-2: without attention pooling; Ours-3: without Transformer self-attention; and Ours: complete model.

## Data Availability

The data presented in this study are available on request from the corresponding author due to ethical reasons related to participant privacy..
